# Supplementary material for: Tunicamycin as a Novel Redifferentiation Agent in Radioiodine Therapy for Anaplastic Thyroid Cancer
Source: Int J Mol Sci. 2021 Jan 22;22(3):1077. doi: 10.3390/ijms22031077 (PMC7865976; doi:10.3390/ijms22031077)
Supplement: Supplementary file 1 [file ijms-22-01077-s001.pdf]

Supporting information

Title: Tunicamycin as novel redifferentiation agent for radioiodine therapy of anaplastic thyroid cancer

Running title: Radioiodine therapy using tunicamycin

Yoon Ju Choi[1,7], Jae-Eon Lee[2], Hyun Dong Ji[1,7], Bo-Ra Lee[2], Sang Bong Lee[3], Kil Soo Kim[2,4], In-Kyu Lee[5,5], Jungwook Chin[6], Sung Jin Cho[6,8], Jaetae Lee[1, 8], Sang-Woo Lee[1,8, *], Jeoung-Hee Ha [7, *], Yong Hyun Jeon[2,8*]

[1]Department of Nuclear Medicine, School of Medicine, Kyungpook National University, Daegu, Korea

[2]Laboratory Animal Center, Daegu-Gyeongbuk Medical Innovation Foundation, Daegu, South Korea.

[3]Korea Institute of Medical Microrobotics (KIMIRo), Gwangju, 61011, South Korea

[4]College of Veterinary Medicine, Kyungpook National University, Daegu 41566, Korea

[5]Department of Internal Medicine, School of Medicine, Kyungpook National University, Daegu, South Korea

[6]New Drug Development Center, Daegu-Gyeongbuk Medical Innovation Foundation, Daegu, South Korea

[7]Department of pharmacology, School of Medicine, Kyungpook National University, Daegu, Korea

[8]Leading-edge Research Center for Drug Discovery and Development for Diabetes and Metabolic Disease, Kyungpook National University Hospital, Daegu, Korea

Supporting Materials and Methods

In vitro bioluminescence imaging

At 24h and 48h after TM treatment, D-luciferin solution was added to treated cells and *in vitro* BLI was acquired immediately after substrate injection using the IVIS Lumina III imaging system (Caliper, CA, USA). Grayscale photographic images and bioluminescent color images were superimposed using LIVINGIMAGE (version 2.12, PerkinElmer) and IGOR Image Analysis FX software (WaveMetrics, Lake Oswego, OR). BLI signals are expressed in units of photons per cm^2 per second per steradian ($P/cm^2/s/sr$).

^{18}F-FDG Uptake Assay

The cells seeded in 24-well plate were treated with TM for 24 h at the concentration of 0, 6, and 12 μM. After aspirating drug-containing medium, cells were washed with 1mL HBSS (Hank's balanced salt solution) and incubated with 500μL HBSS containing 0.5% bovine serum albumin (bHBSS), 74 kBq of ^{18}F-FDG per milliliter for 30 min at 37°C. The cells were then washed twice with ice-cold bHBSS and were lysed with 500μl of 2% sodium dodecyl sulfate (SDS). The radioactivity was measured using a gamma counter. The radioactivity of the cells was normalized using total protein concentrations determined by a BCA kit (Pierce Protein Biology).

Quantitative RT-PCR

Total RNA was extracted using Trizol (Invitrogen, Carlsbad, CA) and reverse transcription reaction was performed on 2 μg of total RNA using RevertAid First Strand cDNA Synthesis Kit (Thermo Scientific, Pittsburgh, PA). Quantitative RT-PCR was carried out with SYBR

Green PCR master mix (Applied Biosystems, Foster City, CA) using a ViiA 7 Real-Time PCR System instrument (Applied Biosystems) with the following primer sets:

NIS (forward, 5’- CTG CCC CAG ACC AGT ACA TGC C-3’; reverse, 5’- TGA CGG TGA AGG AGC CCT GAA G-3’), TSHR (forward, 5’- ACC CTG ATG CCC TCA AAG AGC’; reverse, 5’- GCT TCA GTG TCA AGG TTT CAT TGC’), TPO (forward, 5’- CCT CTG CAA AGA TGT GAA CGA’; reverse, 5’-TCC CGG AGT CTA CGC AGG TT’), TG (forward, 5’- TCT AAC CGA TGC TCA CCT CTT CTG’; reverse, 5’ AGA TGA TGG CAC CTC CTT GAA CC’), and acidic ribosomal protein 36B4 (forward, 5’-CCA CGC TGC TGA ACA TGC T -3’; reverse, 5’- TCG AAC ACC TGC TGG ATG AC -3’). The target genes were normalized to the endogenous reference gene 36B4, and relative mRNA expression levels were calculated in the test and control samples.

Western blot

The cells were treated with or without TM for 24 h and washed twice with cold PBS and lysed with radioimmunoprecipitation assay buffer containing complete protease inhibitor cocktail (Roche). In case of plasma membrane protein for NIS, samples were prepared with a protein biotinylation kit (EZ-Link sulfo-NHS-biotin; Thermo Scientific) according to the manufacturer’s instructions. Briefly, either untreated or treated cells were washed twice with ice-cold PBS/CM (PBS containing 0.1 mM calcium chloride and 1 mM magnesium chloride, pH 7.3) and incubated with EZ link NHS-sulfo-SS-biotin (1 mg/mL) in PBS/CM for 30 min at 4°C. The reaction was quenched by 2 washes with cold 100 mM glycine in PBS/CM and further incubation with 100 mM glycine in PBS/CM at 4°C for 20 min. The cells were then quickly washed 2 times with PBS/CM before lysis with radioimmunoprecipitation assay buffer containing a protease inhibitor cocktail and phosphatase inhibitors (Roche) for 1 h at 4°C with

constant shaking. Lysates were centrifuged at 16,000g for 30 min at 4°C. A portion of the supernatant was used for total cell protein immunoblots. The remaining sample was used to obtain membrane protein by incubation with 100 mL of streptavidin beads (Thermo Scientific) for 1 h at room temperature. The beads were washed 3 times with radioimmunoprecipitation assay buffer, and bound proteins were eluted with 50 mL of Laemmli buffer (62.5 M Tris, pH 6.8; 20% glycerol; 2% sodium dodecyl sulfate; 5% b-mercaptoethanol; and 0.01% bromphenol blue) for 30 min at room temperature. Equal amounts of total and biotinylated plasma membrane protein were loaded in each lane and resolved by 4%– 12% gradient Bis-Tris gel (Invitrogen). Proteins were transferred to 0.2-mm polyvinylidene fluoride membrane (Invitrogen). Membranes were incubated overnight at 4°C with primary mouse monoclonal human NIS–specific antibody (dilution, 1:1,000, Thermo Scientific, catalog no. MS-1653-P1, clone: FP5A), followed by incubation with horseradish peroxidase–conjugated secondary antibody at room temperature. ECL-Plus (Amersham Pharmacia) was used to detect peroxidase activity according to the manufacturer's protocol. Similarly, for other proteins also, equal amounts of protein were loaded in each lane and resolved by 4%–12% gradient Bis-Tris gel (Invitrogen). Proteins were transferred to a 0.2-mm polyvinylidene fluoride membrane (Invitrogen). The membranes were incubated overnight at 4°C with human TSHR, TPO, thyroglobulin (TG), and glucose transporter 1 (Glut1) antibody (Santacruz) and then with the appropriate horseradish peroxidase– conjugated secondary antibody at room temperature. ECL-Plus was used to detect peroxidase activity according to the manufacturer's protocol. Band densities were determined by ImageJ software.

Biodistribution with radioactive ^{125}I

For biodistribution analysis, organs including tumors, liver, lung, heart, kidney, intestine, and others were removed, weighed, and tested for radioactivity using a gamma counter. The results were expressed as the percentage of injected dose per gram of tissue (%ID/g).

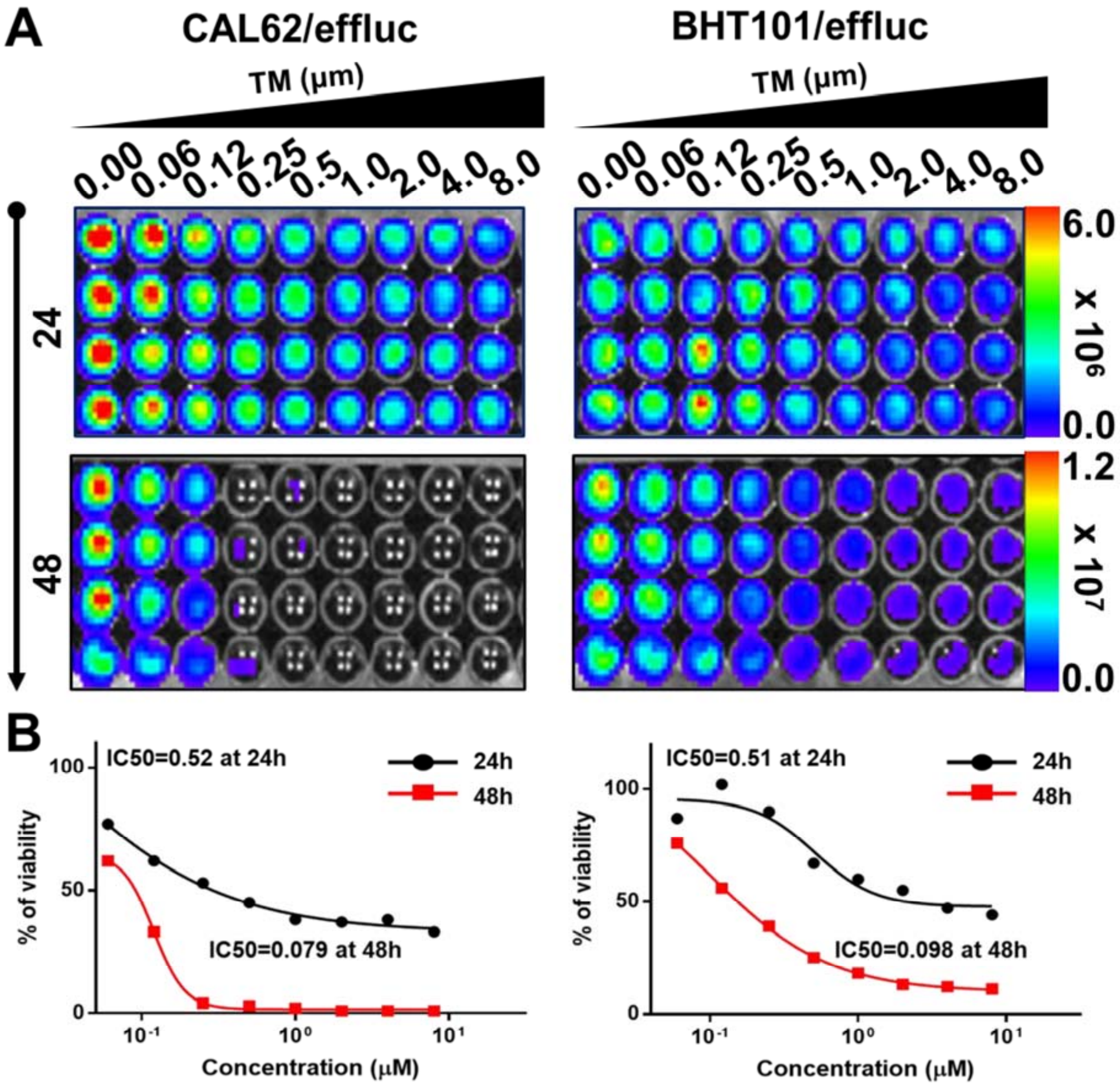

FIGURE S1. Cell proliferation analysis of tunicamycin (TM)-treated ATC cells using *in vitro* BLI. **(A)** *In vitro* BLI of cell proliferation **(B)** and relative viability (%) in TM-treated CAL62 cells and BHT101 cells for 24 h and 48 h. Experiments were performed at least in triplicate, and the mean values ± SD were plotted.

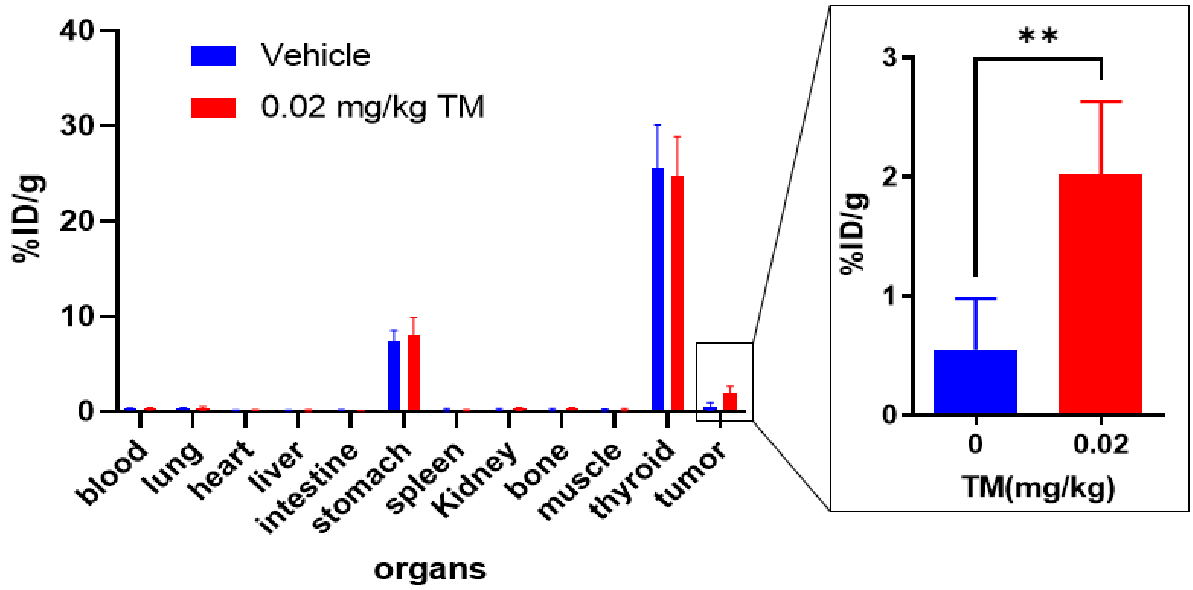

Figure S2. Biodistribution study. When tumor mass was detectable by palpation and inspection, BHT101 tumor-bearing mice received 0.02 mg/kg TM via intraperitoneal injection twice a week for 2 weeks, followed by 1.85 MBq mCi ^{125}I treatment intravenously. For biodistribution analysis, organs including tumors, liver, lung, heart, kidney, intestine, and others were removed, weighed, and tested for radioactivity using a gamma counter. The results were expressed as the percentage of injected dose per gram of tissue (%ID/g). **p<0.005, compared with vehicle.

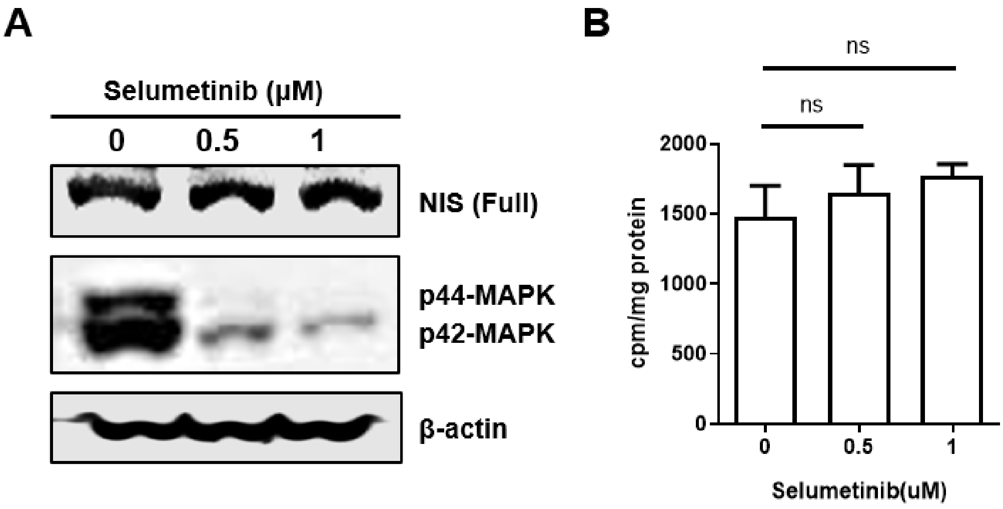

Figure S3. Effect of selumetinib on iodide uptake in BHT101 cells. (A) BHT101 cells were treated with various doses of selumetinib for 24 h. (A) Levels of NIS and p-ERK levels, as well as (B) iodide uptake avidity, was evaluated. N.S, not significant. Data are the mean ± SD of three samples per group.

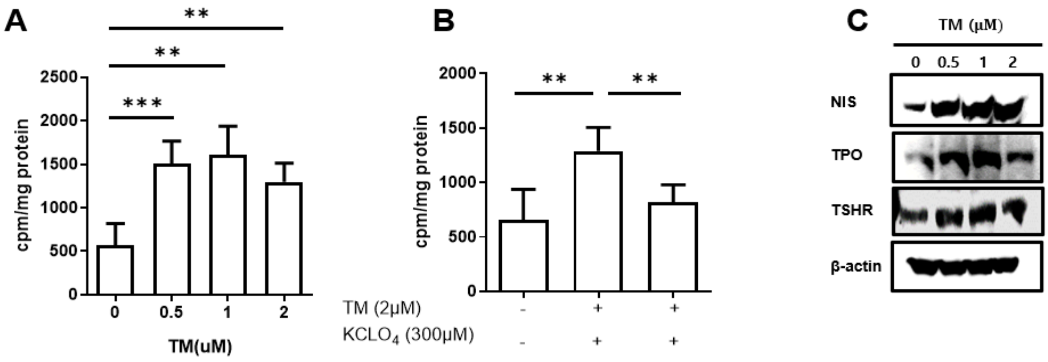

Figure S4. Effect of tunicamycin (TM) on iodide uptake in BCPAP cells. (A) BCPAP cells were treated with various doses of TM for 24 h, followed by iodide uptake assays. (B) To examine the inhibition of iodide uptake, cells were treated with 2 μM TM and 300 μM $KClO_4$ (NIS-specific inhibitor) for 24 h, and iodide uptake was assessed. **, $p<0.005$, ***, $p<0.0005$, compared to absence of TM (Iodide uptake assay). **, $p<0.005$ compared to TM ($KCLO_4$ inhibition study). Data are the mean ± SD of three samples per group.
